# A Comparative Study to Decipher the Structural and Dynamics Determinants Underlying the Activity and Thermal Stability of GH-11 Xylanases

**DOI:** 10.3390/ijms22115961

**Published:** 2021-05-31

**Authors:** Jelena Vucinic, Gleb Novikov, Cédric Y. Montanier, Claire Dumon, Thomas Schiex, Sophie Barbe

**Affiliations:** 1 Toulouse Biotechnology Institute (TBI), Université de Toulouse, CNRS, INRAE, INSA, ANITI, 31400 Toulouse, France; vucinic@insa-toulouse.fr (J.V.); gnovikov87@gmail.com (G.N.); montanie@insa-toulouse.fr (C.Y.M.); cdumon@insa-toulouse.fr (C.D.); 2 Université Fédérale de Toulouse, ANITI, INRAE, UR 875, 31326 Toulouse, France; thomas.schiex@inrae.fr

**Keywords:** GH-11 xylanase, molecular dynamics simulations, enzyme thermostability, enzyme activity, dynamic cross correlation, free-energy landscape, enzyme–substrate interaction

## Abstract

With the growing need for renewable sources of energy, the interest for enzymes capable of biomass degradation has been increasing. In this paper, we consider two different xylanases from the GH-11 family: the particularly active GH-11 xylanase from *Neocallimastix patriciarum*, *Np*Xyn11A, and the hyper-thermostable mutant of the environmentally isolated GH-11 xylanase, *Ev*Xyn11^TS^. Our aim is to identify the molecular determinants underlying the enhanced capacities of these two enzymes to ultimately graft the abilities of one on the other. Molecular dynamics simulations of the respective free-enzymes and enzyme–xylohexaose complexes were carried out at temperatures of 300, 340, and 500 K. An in-depth analysis of these MD simulations showed how differences in dynamics influence the activity and stability of these two enzymes and allowed us to study and understand in greater depth the molecular and structural basis of these two systems. In light of the results presented in this paper, the thumb region and the larger substrate binding cleft of *Np*Xyn11A seem to play a major role on the activity of this enzyme. Its lower thermal stability may instead be caused by the higher flexibility of certain regions located further from the active site. Regions such as the *N*-ter, the loops located in the fingers region, the palm loop, and the helix loop seem to be less stable than in the hyper-thermostable *Ev*Xyn11^TS^. By identifying molecular regions that are critical for the stability of these enzymes, this study allowed us to identify promising targets for engineering GH-11 xylanases. Eventually, we identify *Np*Xyn11A as the ideal host for grafting the thermostabilizing traits of *Ev*Xyn11^TS^.

## 1. Introduction

With the increasing need for renewable and sustainable sources of fuels and chemicals that could help reduce pollution and global warming caused by industrial activities, the importance of biomass-degradation-capable enzymes in biorefinery processes has been rapidly increasing. Endo-1,4-β-xylanases catalyze the hydrolysis of the β-1,4 glycosidic linkage of the xylane backbone in heteroxylans (constituting the lignocellulosic plant cell wall) and produce mainly xylobiose and, to a lesser extent, short xylo-oligosaccharides (XOS) [1]. Xylanases are widely used in industrial processes including pulp and paper, food, and animal feed [2]. Given their ability to contribute to the degradation of hemicellulose and specifically arabinoxylans—the second most abundant renewable biomaterial available after cellulose inside lignocellulosic polymers—their importance in biorefinery processes is rapidly increasing [3]. The development of such effective and competitive bioprocesses requires enzymes that remain highly active under industrial conditions, notably at high temperature. Understanding the molecular basis underlying the thermostability and activity of xylanases is thus of paramount interest for optimizing their properties and meeting industrial constraints.

According to the sequence identity and three-dimensional structure homology, xylanases are classified within the Carbohydrate Active Enzymes database (CAZy) [4] into Glycoside Hydrolase (GH) families 5, 8, 10, 11, 30, and 43. In this paper, we focus our interest on the xylanases from the GH-11 family. The three-dimensional structure of GH-11 xylanases has been compared to the shape of a partially closed right hand and different elements such as fingers, thumb, and palm, have been named accordingly. The fold has a highly conserved β-jelly roll architecture [3] and is composed of two antiparallel β-sheets (β-sheets A and B), which form the fingers of the hand and a single α-helix packed under the β-sheet B, which forms the palm together with a part of the twisted β-sheet B. Although the three-dimensional structure of an enzyme can help in deciphering enzyme function, their dynamic nature is an important feature to consider in enzyme catalysis. Indeed, their thermodynamic and kinetic properties define the conformational states they are likely to adopt and the energy necessary to switch between these respective conformational states. Analysis of the enzyme dynamics has proven to be of great importance for understanding the interplay between their structural features and their specific properties [5]. Molecular dynamics (MD) simulation based methods are highly useful for investigating protein structural dynamics in order to understand the molecular basis of protein recognition, assembly, properties, and activity [6,7,8,9,10]. MD simulation studies of GH-11 xylanases have shown that an increased temperature induces a significant change in the dynamics of the thumb region [11]. This study has revealed that the thumb loop and the palm regions separate at higher temperatures, going from a closed conformation at lower temperature to an open one, possibly facilitating substrate access to the active-site cleft. Similar works showed that this conformational change was also dependent on the presence of the substrate [12,13,14]. MD simulations have been also used to analyze differences in stability between the twelve members of GH-11 xylanases, including thermophile and mesophile ones [15]. Intramolecular hydrogen bonds and salt bridges have been analyzed and revealed to be an important factor, explaining different thermostabilities of two structurally similar xylanases [16]. When investigating enzymes’ structural basis of catalysis and other biochemical properties, one needs to characterize functional molecular motions that may occur on a wide range of timescales and understand their contribution to enzyme functions. Previously reported MD studies on GH-11 xylanases provide useful information about conformational changes that may occur in the nanosecond range, but the timescale of these simulations (a few tens of ns) is still far from the time needed for biological events to occur.

This paper is therefore devoted to a study that aims at investigating the structure–dynamics–activity relationship of GH-11 xylanases using long MD simulations. We focus on two distinct contrasting enzymes of the GH-11 xylanases family. The first one is a particularly active mesophilic GH-11 xylanase from *Neocallimastix patriciarum* (*Np*Xyn11A) [17]. The second one is a less active but hyper-thermostable mutant of an environmentally isolated GH-11 xylanase, *Ev*Xyn11^TS^ [18]. By comparing the structural and dynamic properties of these two enzymes, our main objective is to identify a set of unique characteristics that could explain their respectively high thermostability and activity. To fulfill this objective, 1 μs MD simulations of the free enzymes and in complex with xylohexaose were performed at 310 K and 340 K. MD simulations at 310 K were used to compare the behavior of the two enzymes at a temperature where they are both known to be active and stable. In order to intensify conformational sampling and observe the impact of higher temperature on the mesostable *Np*Xyn11A and thermostable *Ev*Xyn11^TS^, we additionally performed MD simulations at 340 K. Finally, shorter MD simulations at very high temperature (500 K) were also carried out to compare the thermal resistance of these two enzymes and possibly observe details of the initial unfolding process.

Our analysis of these two enzymes reveals contrasting images. *Ev*Xyn11^TS^ appears to be a tightly packed and mostly rigid enzyme owing to its dense hydrogen bonds network enhanced by very stable salt bridges but a relatively narrow and short catalytic cleft combined with a very mobile thumb. Instead, *Np*Xyn11A is less packed with a less dense hydrogen bond network and less stable salt bridges. Its wider catalytic cleft accommodates several well-organized interaction subsites, combined with a more rigid thumb. Thanks to these results, a feasible strategy for the improvement of GH-11 xylanases for biomass degradation emerges.

## 2. Materials and Methods

### 2.1. Molecular Modeling and Molecular Dynamics Procedures

MD simulations of ligand-free enzymes and enzyme–xylohexaose complexes were performed at 310 K, 340 K, and 500 K using the AMBER 18 suite of programs and its GPU pmemd.CUDA version [19,20,21,22] that supports the mixed scaling of 1–4 nonbonded electrostatic and van der Waals terms in amino acids and sugars.

The high-resolution structures of the particularly active GH-11 xylanase from *Neocallimastix patriciarum*, *Np*Xyn11A (PDB code: 2C1F) [17], and the thermostable mutant of the environmentally isolated GH-11 xylanase, *Ev*Xyn11^TS^ (PDB code: 2VUL) [18], were used as starting models for MD simulations. A xylohexaose molecule (X6) was manually docked in the binding cleft of *Np*Xyn11A and *Ev*Xyn11^TS^, using the X-ray structure of the E177Q catalytic acid/base mutant of the xylanase from *Trichoderma reesei* as a template, cocrystallized with xylohexaose (X6) (PDB code: 4HK8) [23].

In enzyme–xylohexaose complexes, the catalytic acid/base residue (Glu201 for *Np*Xyn11A and Glu180 for *Ev*Xyn11^TS^) was protonated. MD simulations were performed for the free enzymes and enzyme–X6 complexes with the AMBER ff14SB force-field [24] for enzymes, and the GLYCAM_06j-1 force field [25] for the xylohexaose substrate. To obtain a neutral charge of the simulated systems, a number of counterions were included. Each enzyme or enzyme–xylohexaose complex together with the counterions was solvated with TIP3P water molecules, using an octahedral box [26] with a minimum distance of 10 Å between the solute and the simulation box edges.

Preparation of simulations consisted of several energy minimization steps (steepest descent and conjugate gradient methods), a gradual heating to the targeted temperature under constant volume over a period of 100 ps followed by equilibration under a constant pressure (1 bar) over 100 ps. Harmonic potential restraints of 25 kcal/mol/Å^2^ were first applied on the solute atoms and gradually removed along the MD preparation schedule. The production phase of simulations in NPT were then carried out at a constant temperature over 1 μs for 310 K and 340 K simulations, and over 100 ns for 500 K simulations. The temperature and the pressure were controlled using the Berendsen algorithms [27]. Long-range electrostatic interactions were handled by using the particle-mesh Ewald method [28]. A 9 Å cut-off for nonbonded interactions was used. The integration time-step of each simulation was 2 fs and the SHAKE algorithm was used to constrain the lengths of all chemical bonds involving hydrogen atoms to their equilibrium values [29]. Atomic coordinates for each simulation were saved every 10 ps.

### 2.2. 3D Structure and Molecular Dynamics Trajectory Analyses

The Amber18-implemented CPPTRAJ module [30] was used to compute several structural and geometrical properties and to perform dynamic cross correlations and principal component analysis from MD trajectories. The salt bridges occurring along MD simulations were detected using the “saltbr” plugin within VMD [31]. Geometrical and topological properties of each enzyme’s active site were measured using the CASTp 3.0 web-server [32]. The PLIP web-server was used to investigate the enzyme–xylohexaose interactions [33].

#### 2.2.1. Structural and Geometrical Properties

For each enzyme, the residues composing the active site were identified and the negative volume (the space encompassed by the atoms that form the active site) and the surface area of the active site was computed.

The root mean square deviation (RMSD) of backbone atoms relative to the starting structure was calculated for each enzyme (in free form or in complex with X6) along each MD simulation. Per-residue B-factors averaged over the entire trajectories were derived from the respective root mean square fluctuations (*RMSF*) calculated on all backbone atoms. *RMSF* calculations provide a crude estimation of the average atomic positional fluctuations over the course of a given MD simulation trajectory. Prior to *RMSF* calculations, the MD snapshots were RMS-fitted onto the average structure to remove all degrees of translations and rotations. The mass-weighted fluctuations of the backbone atoms (*C*, Cα, and *N*) and *B*-factors for each residue were calculated as follows:

(1) $$RMSF=\sqrt{\frac{1}{nsteps}\sum_{i=1}^{nsteps}{∥r_{i}\left(t\right)-\langle r_{i}\rangle ∥}^{2}}$$

and

(2) $$B-\mathrm{factor}=RMSF^{2}\left(\frac{8}{3}\right)\pi^{2},$$

where $r_{i}$ is the position of atom *i* at time *t* and $\langle r_{i}\rangle$ is the average position of the atom. For comparative purposes, given that the two studied enzymes differ in their amino acid sequence length, the calculated B-factor values were matched between the two enzymes by aligning their respective sequences.

Hydrogen bonds (HBs) formed between donor and acceptor atoms in each MD snapshot were detected using the following geometric criteria: the distance from a donor heavy atom D and an acceptor heavy atom A is less than 3 Å and the valence angle between A, a donor hydrogen atom H, and D (A-H-D) is greater than $135^{\circ }$. We measured both *dynamic* and *static* HB counts. The static HB count is the average of the number of hydrogen bonds per residue during the simulation, computed as the number of hydrogen bonds observed during the whole MD trajectory divided by the number of snapshots and the sequence length. The dynamic HB count is the per-residue number of hydrogen bonds observed in *at least one* snapshot of the MD trajectory, similarly normalized by the MD simulation and sequence lengths. Enzyme intramolecular HBs and enzyme–solvent HBs were determined from 1000 regularly spaced snapshots taken along the 1 μs MD trajectory of each enzyme, carried out at T310 K and T340 K. Enzyme–substrate HBs were calculated from 1000 snapshots of the first 100 ns of the respective MD trajectories performed at T310 K and T340 K. The number of salt bridges formed over the course of the MD simulations was calculated assuming that a salt bridge is formed when the distance between the oxygen atoms of acidic residues and the nitrogen atoms of basic residues does not exceed 4 Å. Standard errors were calculated using the block average method [34,35].

#### 2.2.2. Dynamic cross Correlation

Dynamic cross correlations are widely used in MD simulation analysis [36] to quantify the correlation coefficients of motions between atoms in molecular structures [37]. In this study, dynamic-cross-correlation matrices were calculated using the Cα atomic coordinates to quantify the correlated motions in the studied enzyme’s backbone and identify potential domain motions over the course of the respective MD trajectories. Cross-correlation elements for Cα atoms of two residues *i* and *j* are defined as

(3) $$C_{ij}=\frac{\langle r_{i}\;\cdot \;r_{j}\rangle -\langle r_{i}\rangle \langle r_{j}\rangle }{\sqrt{\left[\left(\langle r_{i}^{2}\rangle -{\langle r_{i}\rangle }^{2}\right)\left(\langle r_{j}^{2}\rangle -{\langle r_{j}\rangle }^{2}\right)\right]}}.$$

Highly correlated (resp. anticorrelated) motions are identified by $C_{ij}=1$ (resp. $C_{ij}=-1$).

#### 2.2.3. Principal Component Analysis and Free-Energy Landscape Analysis

Principal Component Analysis (PCA) is a usual dimensionality reduction method. In Molecular Modeling, PCA—or Essential Dynamics [38]—is commonly used to capture the most important molecular motions of macromolecules. A covariance matrix is first constructed from the trajectories of a selected set of atoms. Its diagonalization provides the eigenvectors or components (directions of the atomic motions in the conformational space) with corresponding eigenvalues (amplitude of the respective atomic motions). PCA preserves only a small set of largest amplitude components corresponding to the largest amplitude protein motions, also called collective motions [39].

In this study, PCA was performed on all MD trajectories simulated at T310 K and T340 K. Only Cα atoms were considered for the analysis. To remove global proteins rotations and translations, the snapshots of each trajectory were aligned to their calculated average coordinates.

From the PCA output, the Free-Energy Landscape (FEL) of a protein can be derived, using MD simulation as a sampling method that allows the exploration of conformations near the native-state structure [40,41,42]. The FEL was constructed here along the first two Principal Components (PCs) using the following equation:

(4) $$G_{a}=-kT\ln\left(\frac{P(q_{a})}{P_{max}\left(q\right)}\right),$$

where *k* is the Boltzmann constant, *T* is the temperature of the simulation, $P(q_{a})$ is the probability of a state *a*, and $P_{max}\left(q\right)$ is the probability of the most probable state. Considering two PCs, *i* and *j*, the free-energy landscapes were obtained from the joint probability distributions P(i,j) of the system.

### 2.3. Enzymatic Activities and Thermostability

#### 2.3.1. Enzyme Production and Purification

The sequence of the thermostable environmental xylanase *Ev*Xyn11^TS^ [18] was cloned in a pET28a vector between BamHI and EcoRI to provide an *N*-terminal His${}_{6}$-Tag and facilitate the purification. Both *Ev*Xyn11^TS^ and the mesostable *Neocallimastix patriciarum* *Np*Xyn11A [17] were expressed and purified as follows. Briefly, proteins were expressed in *E. coli* BL21 (DE3) and cultured until $OD_{600\;nm}$ reached 1.0 in Terrific Broth at 37 °C, and recombinant enzyme expression was induced by the addition of isopropyl-β-d-thiogalactopyranoside to a final concentration of 1 mM, then further incubated for 4 H. Both proteins were purified as follows: cells were harvested by centrifugation (10 min, 4424× *g*) and pellets were suspended in 50 mM phosphate buffer (pH 7.2) containing 300 mM NaCl. Cell lysis was achieved using a 15-mL tube filled with 2 mL of 0.1 mm zirconia-silicate beads (Cole-Parmer™, Vernon Hills, IL, USA) and homogenized for 40 s at 6.5 m s${}^{-1}$ using a FastPrep-24™ 5G (MP Biomedicals, Irvine, CA, USA). Proteins bearing the His${}_{6}$-Tag were purified by immobilized metal ion affinity chromatography (IMAC), as previously described [43].

#### 2.3.2. Enzyme Assay

The specific activity (SA) of the xylanases was measured using the dinitrosalicylic acid (DNSA) assay, as previously described [43], in a final concentration of 1% (w/v) wheat arabinoxylan (WAX) medium viscosity (Megazyme, Ireland) in 50 mM sodium phosphate, 12 mM sodium citrate pH 6, supplemented with 1 mg/mL of bovine serum albumin (BSA) Sigma. Final concentration of *Ev*Xyn11^TS^ and *Np*Xyn11A is 25 nM and 5 nM, respectively.

#### 2.3.3. Differential Scanning Fluorimetry

The melting temperature ($T_{m}$) was determined using Differential Scanning Fluorimetry (DSF). Samples were loaded into a 96-well PCR plate (Bio-Rad, Hercules, CA, USA) at a final volume of 20 μL. The concentration of protein in each well was 10 μM supplemented with 5× SYPRO Orange (Thermo Fisher Scientific, Waltham, MA USA). DSF experiments were carried out using a CFX96 real-time PCR system (Bio-Rad, Hercules, CA, USA), set to use the 480 nm/500 nm excitation and 560 nm/580 nm emission channels. The samples were heated from 20 °C to 99.5 °C. A single fluorescence measurement was taken every 0.3 °C and each measurement lasted 3 s. $T_{m}$ was given by the inflection point of the curve of relative fluorescence unit (rfu) as a function of temperature (rfu=f(T)). The degree of thermal shift ($\Delta T_{m}$) was calculated as follows: $\Delta T_{m}=T_{m}\left(x\right)-T_{m}^{0}$, with $T_{m}\left(x\right)$ being the $T_{m}$ measured in each condition, and $T_{m}^{0}$ being the $T_{m}$ of reference, measured in the purification buffer.

## 3. Results and Discussion

### 3.1. Biochemical and Structural Properties

Specific activity on wheat arabinoxylan (WAX) and melting temperature ($T_{m}$) of *Np*Xyn11A and *Ev*Xyn11^TS^ were measured in the same conditions. *Np*Xyn11A has an average specific activity of 3917 ± 43 IU mg${}^{-1}$ while that of *Ev*Xyn11^TS^ is 1012 ± 12 IU mg${}^{-1}$. $T_{m}$ values are 59.7 ± 0 °C and 94.9±0.1 °C, respectively.

**Figure 1:**
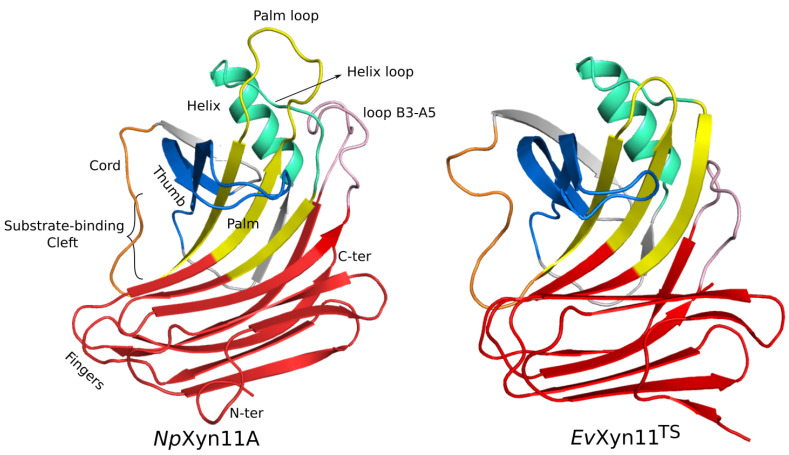
3D structures of *Np*Xyn11A (**left**) and *Ev*Xyn11^TS^ (**right**). Visual representation of the right-hand analogy regions are indicated: the fingers are in red, palm region is in yellow, thumb region is in blue, cord is in orange, helical region is in cyan-green, and the loop B3-A5 is in pink. The substrate binding cleft is also shown.

*Np*Xyn11A and *Ev*Xyn11^TS^ present a β-jelly roll fold as all GH-11 enzyme members. The overall shape of their structure can be described as a partially closed right hand consisting of one domain folding into two β-sheets twisted to form a cleft on one side of the protein where the active site is found. The cleft is covered by a region called the thumb and partially closed on one extremity by a long loop called the cord. Different other molecular regions such as fingers and palm, were named accordingly. The positions of the residues that compose these regions in each enzyme are given in Table 1 and shown in Figure 1.

*Np*Xyn11A is 26 residues longer than *Ev*Xyn11^TS^. As it can be observed in Figure 1, their structure is comprised of β-strands and one α-helix. Loops that connect these secondary structure elements may play an important role on the properties of these enzymes by regulating their structural dynamics. The comparison of the three-dimensional structures of thermostable and mesostable enzymes show that, in most cases, thermostable enzymes tend to have a more compact structure with shorter loops and a more densely packed hydrophobic core [44]. When comparing the 3D structures of *Np*Xyn11A and *Ev*Xyn11^TS^ (Figure 1), we can see that the two enzymes present very different loops, generally longer in *Np*Xyn11A. Especially, the helix, palm, and B3-A5 loops are 8, 12, and 13 residues long in *Np*Xyn11A, compared to 5, 5, and 9 in *Ev*Xyn11^TS^, respectively. The palm loop is particularly long in *Np*Xyn11A compared to any other GH-11 xylanases. Furthermore, the loops connecting the β-strands of the fingers are also longer in *Np*Xyn11A than in *Ev*Xyn11^TS^.

The active site is a long cleft with the catalytic dyad (two glutamic acid) located in the middle, which is in complete agreement with the endo mechanism of GH-11. The cleft comprises a series of subsites, each one capable of binding a xylose moiety. The subsites that bind the glycone and aglycone regions of the substrate are prefixed by − and +, respectively, and annotated with a number related to their proximity to the site of bond cleavage (the glycosidic bond between the xylose residues at the +1 and −1 subsites cleaved by the enzyme). The size of the substrate binding cleft defines the degree of polymerization of xylo-oligosaccharide end products. According to kinetic and structural investigations of GH-11 xylanases, their active sites potentially have up to three (−) subsites and three (+) subsites. In order to investigate the enzyme–substrate interactions at each subsite of the substrate binding cleft, 3D molecular models of a xylohexaose (comprised of 6 xylose units) docked in the cleft of *Np*Xyn11A and *Ev*Xyn11^TS^ were built and investigated by molecular dynamic simulations.

### 3.2. Molecular System Stability

**Figure 2:**
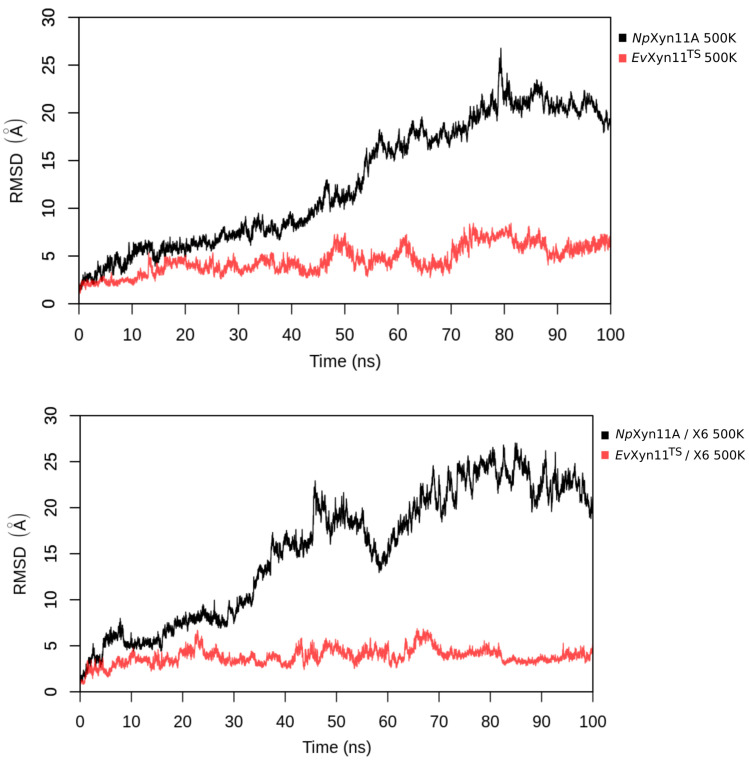
Backbone Root Mean Square Deviation (in Å) of systems in free-enzyme forms and enzyme–substrate complexes at 500 K.

MD simulations were carried out to compare and investigate the mesostable *Np*Xyn11A and the hyper-thermostable *Ev*Xyn11^TS^ in their free and X6-bound forms. The stability of the studied systems at different temperatures was evaluated by monitoring the backbone root mean square deviation (RMSD) as a function of time. This was firstly done for short (100 ns) simulations performed at a very high temperature (500 K) in order to compare the resistance of these two enzymes to thermal denaturation and reveal details of the unfolding process (see Figure 2). The corresponding RMSD time series show a drastic increase in the RMSD of *Np*Xyn11A in the free-enzyme and the enzyme–substrate complex (up to 25 Å. Instead, *Ev*Xyn11^TS^ presents relatively low RMSDs at such high temperature, especially in the enzyme–substrate complex form.

**Figure 3:**
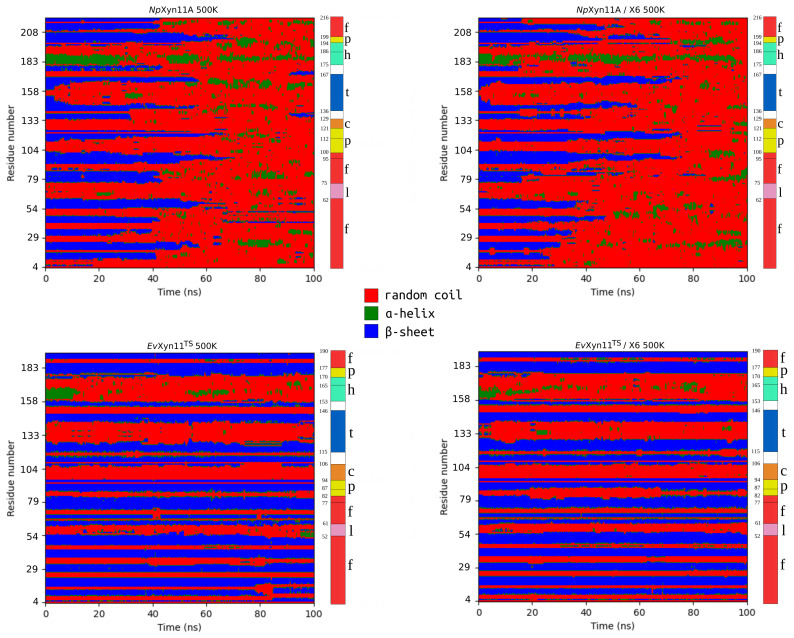
Secondary structure propensities during 100 ns of simulation time for *Np*Xyn11A (**up**) and *Ev*Xyn11^TS^ (**down**) in free-enzymes (**left**) and enzyme–substrate complexes (**right**) at 500 K. On the right of each plot, the (f)inger, (l)oop, (p)alm, (c)ord, (h)elix, and (t)humb regions are indicated, following the colors from Figure 1.

Figure 3 displays the secondary structure propensities over the 100 ns of simulation time. The figure shows a major loss of secondary structure elements in *Np*Xyn11A along the MD simulation: β-strands are transformed in random coils and the alpha-helix is also disrupted. In *Ev*Xyn11^TS^ most of the β-sheets are maintained. These results show the striking difference in structural stability between these two enzymes, which is consistent with the higher thermostability of *Ev*Xyn11^TS^.

**Figure 4:**
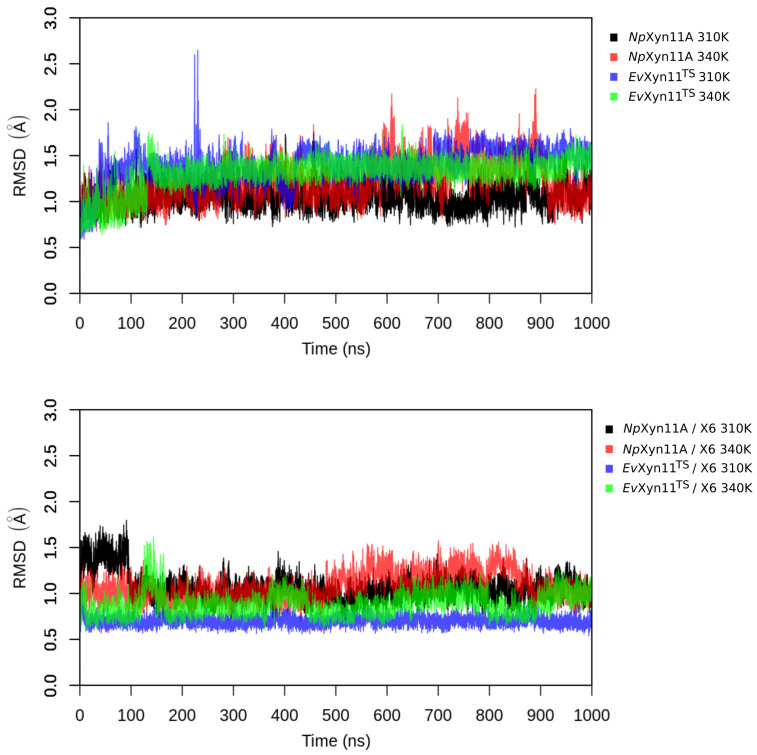
Backbone Root Mean Square Deviation (in Å) of systems in the free-enzyme and enzyme–substrate complex forms at different temperatures.

**Figure A1:**
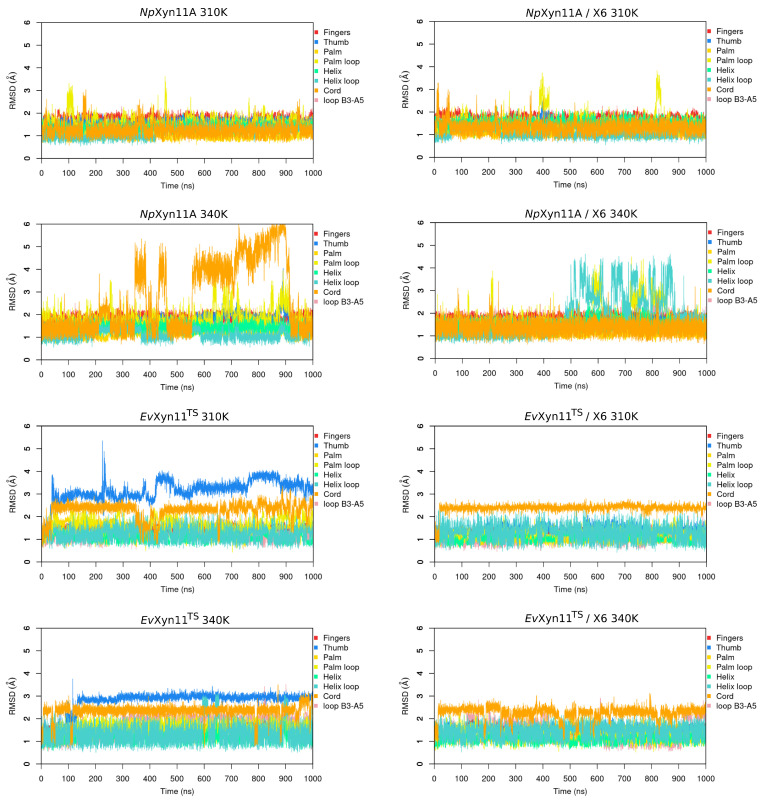
Backbone Root Mean Square Deviation (in Å) of each key region in the free-enzyme and enzyme–substrate complex forms at different temperatures.

RMSD analysis was further carried out on the free enzymes and enzyme–substrate complexes at 310 K and 340 K over 1 μs of simulation time (see Figure 4). In general, we observe fluctuations in RMSD values during the first 200 ns, which are followed by a stabilization marked by a characteristic plateau reaching an equilibrium value between 0.5 and 1.5 Å, depending on the system. However, in the case of *Np*Xyn11A at 340 K, significant fluctuations in RMSD values can still be observed between 600 ns and 900 ns in both free and complex enzyme forms. As the maximum RMSD for all systems does not exceed 2 Å and remains relatively stable over time, one can conclude that the respective systems are stable with respect to the chosen MD parameters. As shown in the RMSD time series, conformational changes were observed over the course of the 1 μs simulation, but the structures did not show any signs of initial unfolding at 340 K. Figure A1 in Appendix A shows key regions RMSD and highlights differences in conformational rearrangements between the two enzymes along MD simulations at 310 and 340 K. In the free *Np*Xyn11A, the largest variations occur in the cord region along MD simulation at 340 K, while in the enzyme–substrate complex, the helical region varies the most. Variations in the RMSD of the palm loop are also observed, especially along the second half of the simulations at 340 K. In the case of free *Ev*Xyn11^TS^, the highest RMSD values and variations are observed in the thumb region. In the complex, the thumb region that covers the substrate binding cleft is stable while the RMSD values remain relatively high in the cord region.

### 3.3. Flexibility Analysis

**Figure 5:**
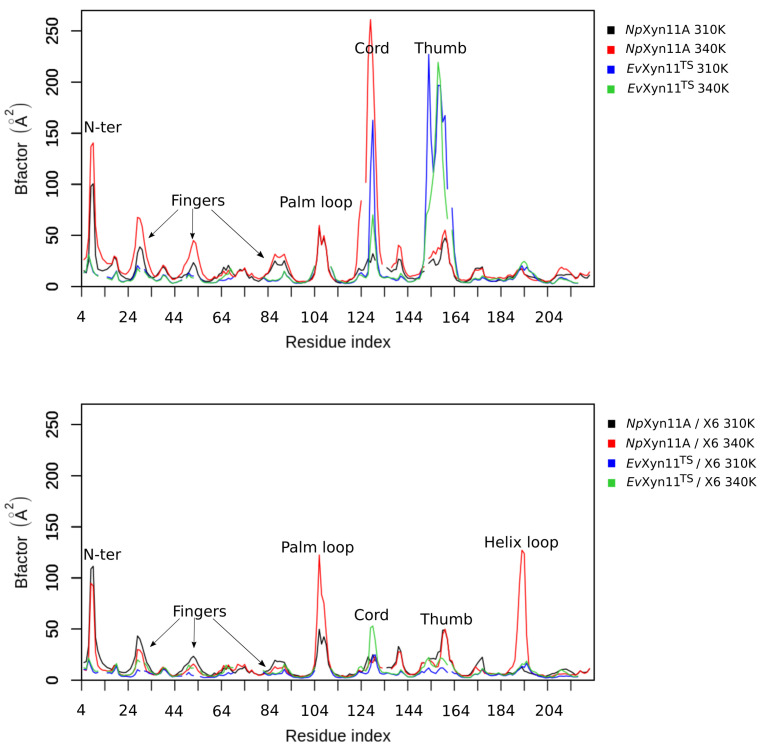
Per-residue average backbone B-factor profiles calculated from MD trajectories of *Np*Xyn11A and *Ev*Xyn11^TS^ in their free-enzyme and enzyme–substrate complex forms at two different temperatures (310 K and 340 K). Gaps in curves correspond to the gaps introduced in the alignment.

In order to compare the backbone flexibility of these two enzymes, per-residue B-factors were calculated and monitored over the course of the simulations from the Root Mean square fluctuations (RMSF) on all backbone atoms. Figure 5 shows the backbone B-factor values as a function of the residue index in an alignment of *Np*Xyn11A and *Ev*Xyn11^TS^, for both the free-enzyme and the enzyme–substrate complexes.

The same backbone B-factor patterns can be observed in both forms of *Np*Xyn11A at 310 K and 340 K, with an expected increase in fluctuations with higher temperature. Overall, *Ev*Xyn11^TS^ exhibits lower backbone B-factor values with smaller fluctuations than *Np*Xyn11A. The B-factor profiles of *Ev*Xyn11^TS^ in the free enzyme and the enzyme–substrate complex are very similar at 310 K and 340 K, except for the thumb region.

In its free form, *Np*Xyn11A has an average B-factor value of 14.7 Å^2^ at 310 K and 23.7 Å^2^ at 340 K, while the free form of *Ev*Xyn11^TS^ has an average B-factor value of 18.2 and 15.5 Å^2^, respectively. In the respective enzyme–substrate complexes, both enzyme backbones tend to be less flexible. For *Np*Xyn11A, the average B-factor values are 13.3 Å^2^ at 310 K and 14.2 Å^2^ at 340 K. For *Ev*Xyn11^TS^ they reduce to only 6.2 and 8.9 Å^2^ at 310 K and 340 K, respectively. One can observe a total of 7 major B-factor peaks in the B-factor profile of the free *Np*Xyn11A. They are located in the *N*-ter, fingers, palm loop, thumb, and cord regions, with the last region showing the highest sensitivity to increased temperature. For the *Np*Xyn11A enzyme–substrate complex, the backbone B-factor values are generally reduced. The thumb and *N*-ter regions present approximately the same flexibility as in the free-enzyme form, at both studied temperatures. The cord, located at one of the extremities of the cleft (at the +3 subsite) is less flexible in the enzyme–substrate complex, even at 340 K. However, in the enzyme–substrate complexes, a B-factor peak appears in the loop located between the α-helix and the β-sheet B4 (between residues 183 and 203, here referred to as the helix loop). The palm loop B-factor is also much higher than in the free-enzyme forms.

Compared to the mesostable *Np*Xyn11A, *Ev*Xyn11^TS^ presents a much lower number of flexible regions. The *N*-ter region as well as the fingers, palm loop, and α-helix loop regions do not present any apparent backbone flexibility in *Ev*Xyn11^TS^. The very low B-factor of the *N*-ter region can be explained by the presence of a disulfide bridge constraining the backbone dynamics in this region. It is well known that disulfide bridges play an important role in the stability of all xylanases of the GH-11 family, and the impact of this disulfide bridge may go beyond the *N*-ter region and play a role on its overall stability. This property was used in the past to engineer thermostable enzymes by introducing the disulfide bridge in the *N*-ter region [45].

Nevertheless, *Ev*Xyn11^TS^ in its free form presents a high mobility of the thumb region in contrast to that of the particularly active *Np*Xyn11A enzyme. In *Ev*Xyn11^TS^-substrate complex, an important reduction of B-factors of the thumb is observed, suggesting that the presence of the substrate stabilizes this region. Instead, the relatively high B-factors of the cord region in both the free-enzyme and enzyme–substrate complex forms of *Ev*Xyn11^TS^ indicate that the binding of the ligand does not completely reduce its overall flexibility.

**Figure 6:**
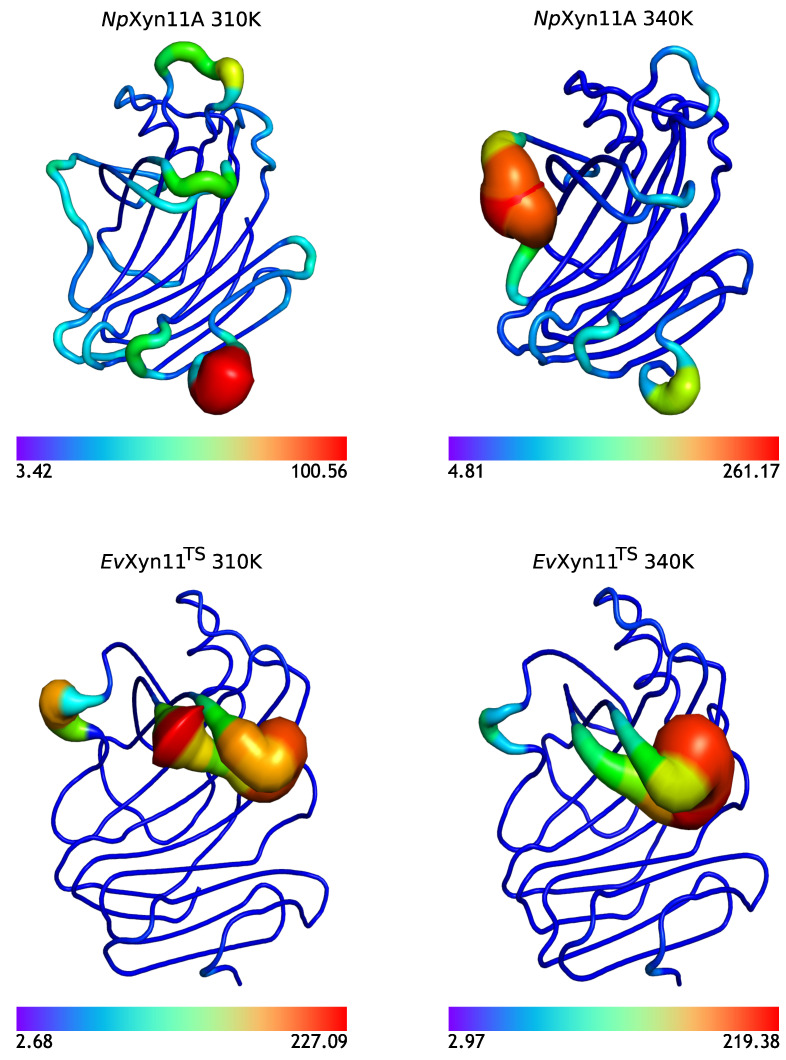
Average backbone B-factor profiles calculated from MD trajectories of *Np*Xyn11A and *Ev*Xyn11^TS^ at two different temperatures (310 K and 340 K), mapped onto their backbone. Note that each image uses a specific scale.

Overall, the flexibility analysis of the mesostable *Np*Xyn11A and hyper-thermostable *Ev*Xyn11^TS^ show that *Ev*Xyn11^TS^ has less flexible regions and is therefore globally less flexible than *Np*Xyn11A. High B-factor values only appear in the thumb region and only in the free-enzyme form (Figure 6). The greater stability of this region in the enzyme–substrate complex can be explained by the presence of the substrate and its important interactions with the thumb. In order to confirm the previous results and obtain more insights into the conformational dynamic of these flexible regions in both enzymes, we compared the dynamic cross correlation of the backbone of their respective 3D structures, in the presence and in the absence of the substrate.

### 3.4. Dynamic cross Correlation

**Figure 7:**
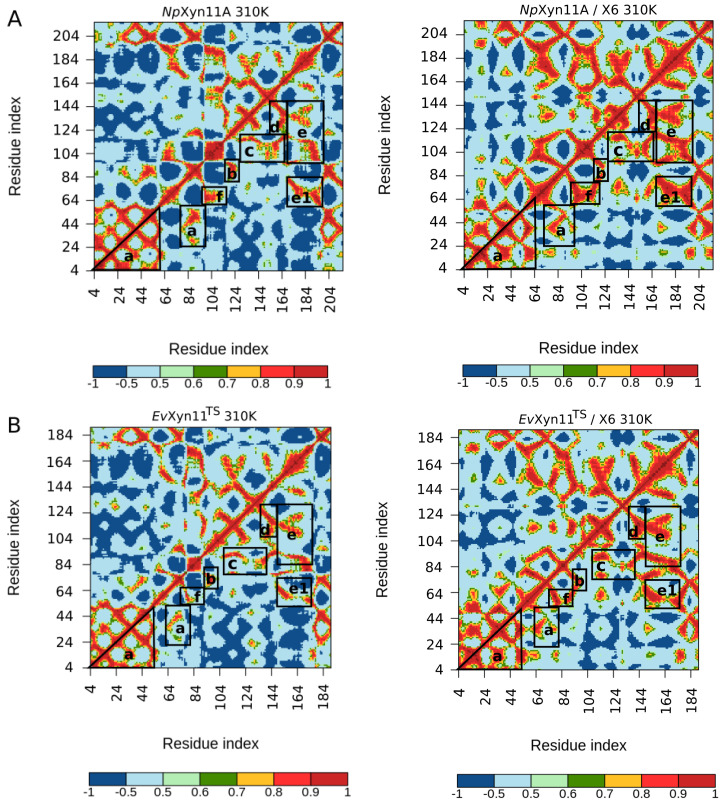
Dynamic cross-correlation analysis from MD trajectories of *Np*Xyn11A (**A**) and *Ev*Xyn11^TS^ (**B**) in their free-enzyme and enzyme–substrate complex forms at 310 K.

**Figure A2:**
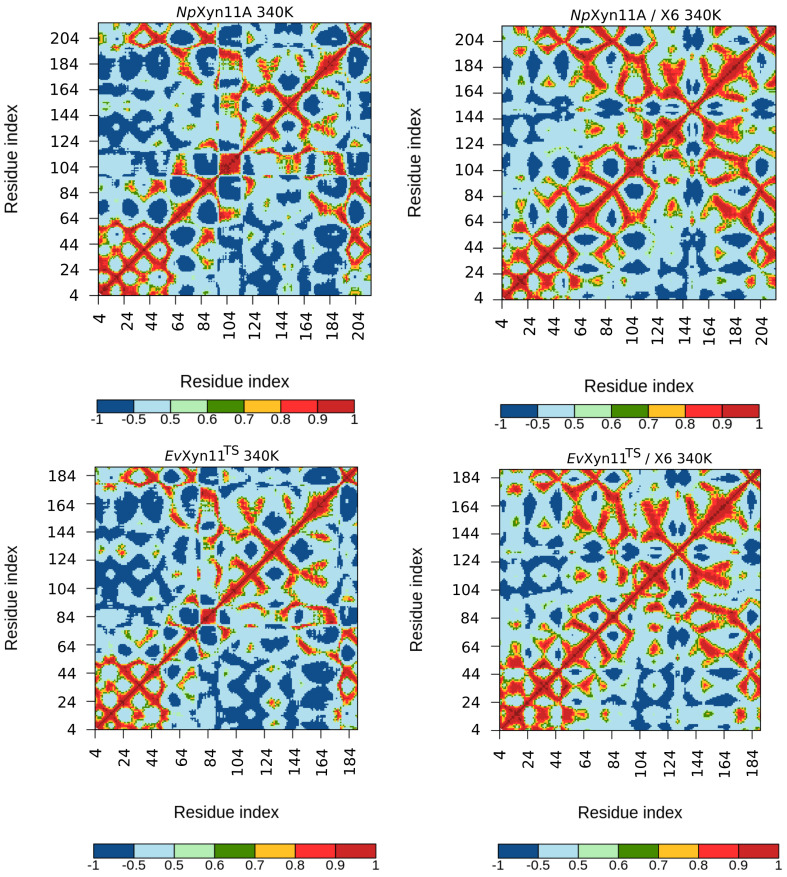
Dynamic cross-correlation analysis from MD trajectories of *Np*Xyn11A and *Ev*Xyn11^TS^ in their free-enzyme and enzyme–substrate complex forms at 340 K.

Dynamically cross-correlated motions were analyzed from MD trajectories at 310 K and 340 K for both enzymes in their free and complex forms (see Figure A2 and Figure 7). Both enzymes exhibit highly similar global dynamics, with very similar regions showing highly correlated motions. The finger regions tend to be dynamically correlated with the *N*-ter region (zone *a* in Figure 7A,B). A correlation of the cord region backbone dynamics with the finger region can be observed in both enzymes (zone *b*). The backbone dynamics of the thumb and palm loop region are highly correlated in *Np*Xyn11A (Figure 7A, zone *c*). This correlation is reduced in *Ev*Xyn11^TS^ (Figure 7B, zone c), probably because of the short palm loop of this enzyme. A correlation between the β-sheet region of the thumb with the cord region and its prolongation can also be noticed (zone *d*). Finally, other correlations involving the helix region and its surroundings can be observed (zone e1 and *e*).

Compared to the free-enzyme forms, the enzyme–substrate complex forms present a higher proportion of positively correlated motions. This is particularly pronounced for the correlations between the loop connecting β-sheets B3 and A5 with the palm loop (zone *f*) and the α-helix region (zone e1), which is specifically increased in the enzyme–substrate complex forms of *Np*Xyn11A (Figure 7A).

In *Ev*Xyn11^TS^, this B3-A5 loop is correlated with the α-helix region (Figure 7B, zone e1), but the correlation with the palm loop is almost nonexistent (Figure 7B, zone *f*). As mentioned previously, this could be caused by the shorter, and therefore less flexible, palm loop of *Ev*Xyn11^TS^.

These results suggest that the palm loop may play an important role in the higher flexibility of *Np*Xyn11A. To validate this hypothesis, this region may be engineered to improve the thermal stability of *Np*Xyn11A.

### 3.5. Free-Energy Landscapes

PCA analysis was based on the first two principal components that explain, on average, 30% of the total protein motions. The contribution of the first principal component varies between 5% and 35%. The minimal value of 5% corresponds to the simulation performed on *Ev*Xyn11^TS^ in complex with xylohexaose at 310 K. This system showed very low B-factor values in the simulations resulting from the noise that spreads equally along all axis.

**Figure 8:**
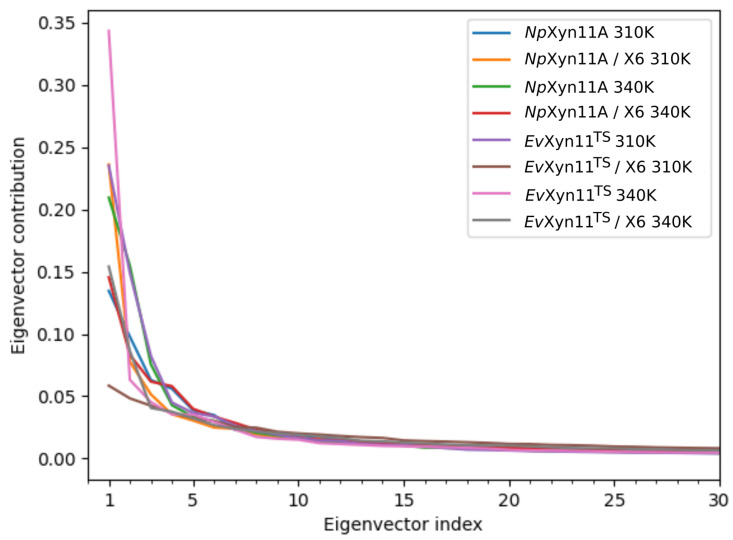
Eigenvector contribution as a function of the eigenvector index. Only the first 30 eigenvectors are shown.

For each frame, the projection of the transformed coordinates along all eigenvectors (PCs) was calculated, and each eigenvector contribution was derived from its respective eigenvalue. Figure 8 shows that the contributions of PCs quickly reduce to 0.

**Figure 9:**
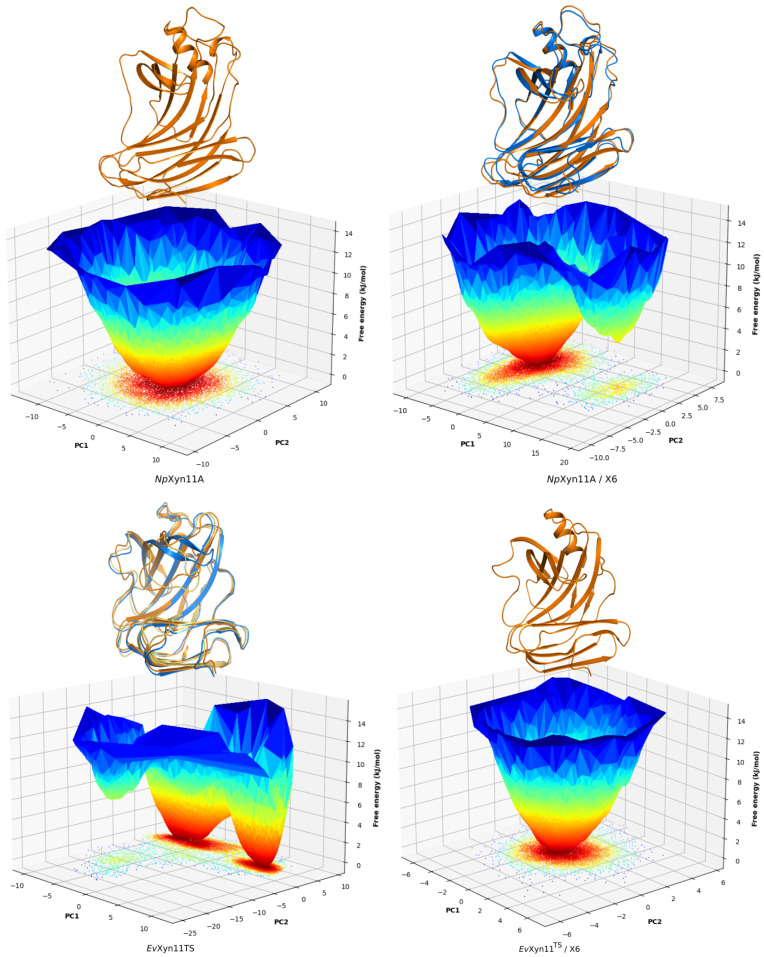
Free-energy landscapes of *Np*Xyn11A and *Ev*Xyn11^TS^ at 310 K in their free-enzyme and enzyme–substrate complex forms as a function of the first (PC1) and second (PC2) eigenvectors. The color bar represents the free-energy values in kJ/mol. The 3D structure corresponding to the global free-energy minimum is displayed in orange cartoon, while the blue one refers to the alternative conformation corresponding to the free-energy minimum of the second basin.

The FELs of *Np*Xyn11A and *Ev*Xyn11^TS^ in their free-enzyme and complex forms at 310 K are shown in Figure 9. Only one free energy basin is observed for *Np*Xyn11A in its free form, indicating the presence of one major ensemble of conformational substates and a general stability of the system. On the bound form, a second basin appears but has high energy, and the two corresponding conformations remain globally similar.

*Ev*Xyn11^TS^ exhibits more conformational sampling in its free-enzyme form, in accordance with the previous B-factor (Figure 5) and per-region RMSD analyses (Figure A1), from which important conformational changes of the thumb region in the free-enzyme form were highlighted. Its free-energy landscape and structures corresponding to its global free-energy minima clearly show multiple stable conformations of the thumb. In the bound form, *Ev*Xyn11^TS^ thumb region is stabilized and a unique basin remains.

**Figure A3:**
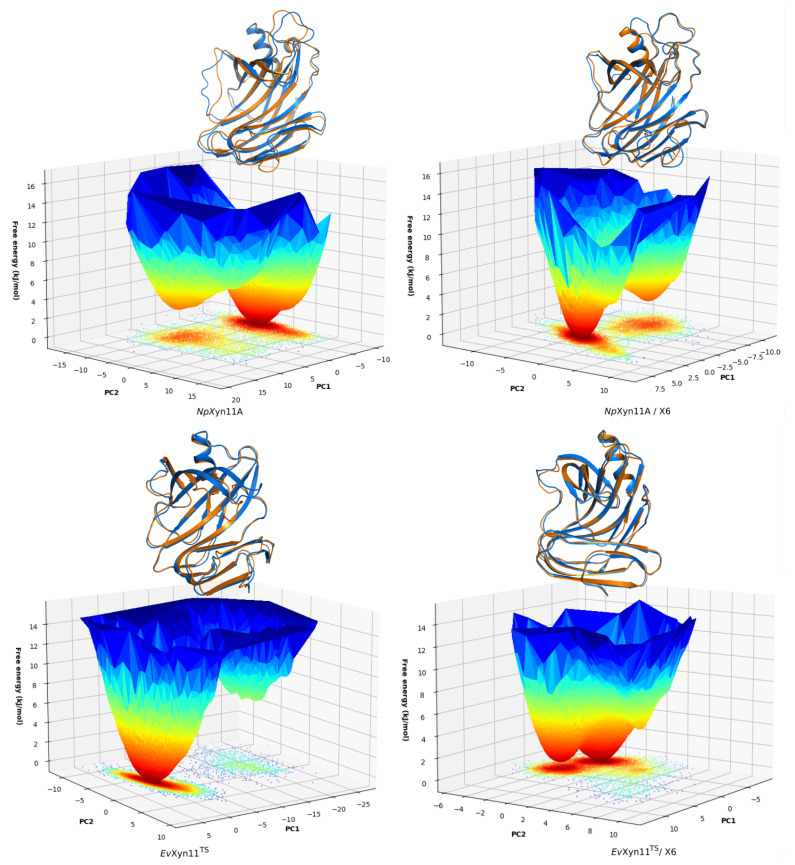
Free-energy landscapes of *Np*Xyn11A and *Ev*Xyn11^TS^ at 340 K in their free-enzyme and enzyme–substrate complex forms as a function of the first (PC1) and second (PC2) eigenvectors. The color bar represents the free energy values in kJ/mol. The 3D structure corresponding to the global free-energy minimum is displayed in orange cartoon, while the blue one refers to the alternative conformation corresponding to the free-energy minimum of the second basin for the enzyme–substrate complexes.

Figure A3 shows the same FEL for the free-enzyme and enzyme–substrate forms at 340 K. At this temperature, multiple basins are observed for both forms of the enzymes, following the diversified conformational sampling. The results confirm the B-factor analysis: the free *Np*Xyn11A variants show differences in the cord region while the enzyme–substrate form changes are mostly observed in the α-helix loop, as the cord region adjusts stably to the substrate with the formation of the +3 subsite.

For the thermostable *Ev*Xyn11^TS^ in free form, the second basin has smaller size, higher energy, and corresponds to important movements of the thumb. When bound and stabilized by its substrate, the scale of movements of *Ev*Xyn11^TS^ is strongly reduced and the two observed variants show little changes in energy or in conformation, with no specific localized change.

#### 3.5.1. Hydrogen Bonds, Salt Bridges, and SASA

The static and dynamic intramolecular and enzyme–solvent hydrogen bond (HB) counts in *Np*Xyn11A and *Ev*Xyn11^TS^ in their free-enzyme and enzyme–substrate complex forms were computed from MD simulations at 310 K and 340 K (Table 2). *Ev*Xyn11^TS^ has more static hydrogen bonds, which contribute to the formation of larger stabilizing interaction networks. *Np*Xyn11A instead possesses a higher number of dynamic HBs, which reflect the dynamic formation of competitive HB interactions. Furthermore, the total number of enzyme–solvent hydrogen bonds observed during 1 μs MD simulation is also higher for *Np*Xyn11A. The transient existence of a greater number of dynamic HBs in *Np*Xyn11A and with the solvent is consistent with its greater flexibility.

**Figure 10:**
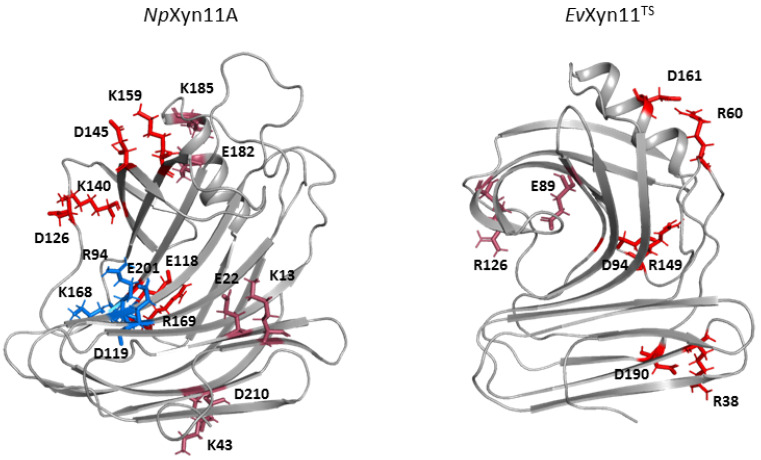
Location of salt bridges in *Np*Xyn11A and *Ev*Xyn11^TS^, colored by their frequency of occurrence (red > 80%, pink between 50% and 80%, and blue < 50%) and calculated from 1 μs MD simulations.

Salt bridges have been identified as one of the main factors contributing to thermostability within the GH-11 family [16,46]. We monitored the different salt bridges that are formed within the protein structures over 1 μs of simulation time (Table 3). The three-dimensional structures of the identified salt bridges in the free-enzymes are shown in Figure 10. In the enzyme–xylohexaose complexes, the same salt bridges as those observed in the unbound enzymes were detected, except the ones involving a catalytic residue, Glu201 (the catalytic acid–base residue in *Np*Xyn11A) or Glu89 (the catalytic nucleophile residue in *Ev*Xyn11^TS^), given that these residues interact with the substrate in the complex.

A total of 8 salt bridges stabilize the *Np*Xyn11A enzyme, against the 4 in the highly thermostable *Ev*Xyn11^TS^. However, salt bridges are present in 33 to 98% of the total simulation time for *Np*Xyn11A, against 94 to 99% of the total simulation time for *Ev*Xyn11^TS^.

In *Np*Xyn11A, a relatively stable salt bridge (80% occurrence frequency) is formed by Asp145–Lys159 in the important thumb region. This may explain the moderate flexibility of this region in *Np*Xyn11A at both studied temperatures and suggests that in both bound and unbound forms, *Np*Xyn11A possesses a stable thumb conformation, which is probably already competent for catalysis. On the contrary, the absence of an intrathumb salt bridge in *Ev*Xyn11^TS^ can explain the high flexibility of this region observed in this enzyme.

In *Np*Xyn11A, another salt bridge is formed by the Asp126–Lys140 pair. Located between the cord and the thumb region, this salt bridge becomes less frequent at 340 K, which is consistent with the increased backbone flexibility of the cord region in the free-enzyme form of *Np*Xyn11A at 340 K (Table 3). Analysis of salt bridges also reveals that the stable Glu118–Arg169 (in *Np*Xyn11A) and Asp94–Arg149 (in *Ev*Xyn11^TS^) interactions occupy the same location when superimposing the structures, meaning that these salt bridges are conserved in both xylanases.

In *Np*Xyn11A, an intrahelix salt bridge is also found between the residues Glu182 and Lys185 with an occurrence frequency of 73%. Other salt bridges were detected with a lower occurrence frequency, especially the Glu22–Lys13 (45% occurrence frequency) and the Lys42–Asp210 (56% occurrence frequency) salt bridges located between the fingers. These salt bridges are less stable than the Arg38–Asp190 salt bridge (99% occurrence frequency) found in *Ev*Xyn11^TS^ fingers. The presence of both this salt bridge and a disulfide bridge must strongly contribute to the high stability of the *N*-terminal and fingers regions of *Ev*Xyn11^TS^. In *Ev*Xyn11^TS^, a very stable (98% frequency) salt bridge, formed by the Asp161–Arg60 pair between the α-helix and the B3-A5 loop (in pink in Figure 1) must also contribute to the global stability of the enzyme by stabilizing the interaction between two distinct regions of the 3D structure involving a surface loop.

Overall, the formation of very stable salt bridges in *Ev*Xyn11^TS^ probably plays an essential role for structural maintenance and, consequently, for the enhanced thermostability of the protein.

Finally, Table 4 shows the average Surface Accessible Solvent Areas for each enzyme over the course of their MD trajectories. *Np*Xyn11A shows higher SASA than *Ev*Xyn11^TS^, which is consistent with the higher number of enzyme–solvent hydrogen bonds detected in *Np*Xyn11A Ȧll of these analyses indicate that the mesostable *Np*Xyn11A enzyme is less tightly packed than the hyper-thermostable *Ev*Xyn11^TS^ enzyme, and possesses a lower number of static intrahydrogen bonds and less-stable salt bridges.

#### 3.5.2. Analysis of Enzyme/Substrate Interactions

**Figure 11:**
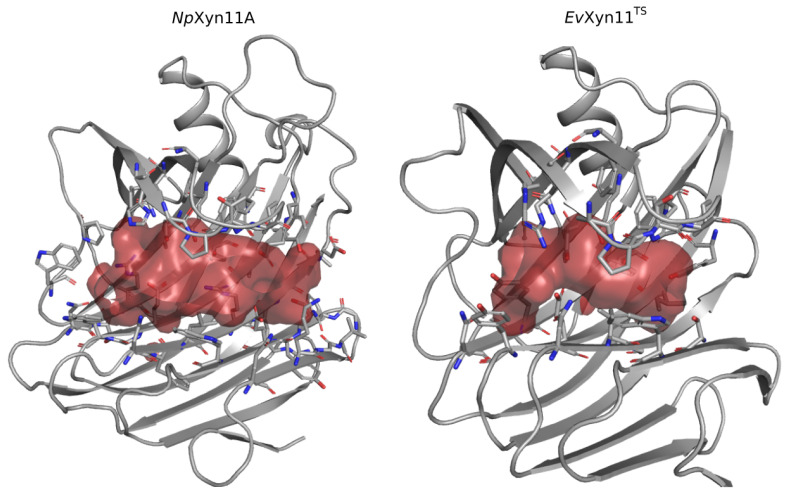
Volume of enzymes active site cleft in *Np*Xyn11A and *Ev*Xyn11^TS^.

One of the main features of the globular structure of these enzymes is the presence of a long cleft located in the center of the enzyme, which contains the active site (also shown in Figure 1). The active site of each enzyme was analyzed in terms of residue composition, negative volume, and area of the open cleft. Figure 11 shows the residues that compose each enzyme’s active site as well as its corresponding negative volume (summarized in Table 5 with the corresponding area).

In the center of the active site of each enzyme are the two conserved catalytic residues, the catalytic nucleophile (Glu113 in *Np*Xyn11A and Glu 89 in *Ev*Xyn11^TS^) and the catalytic acid–base (Glu201 in *Np*Xyn11A and Glu181 in *Ev*Xyn11^TS^). Moreover, several other amino acids in the binding site of both enzymes are highly conserved in GH-11 xylanases [3,47], including Ile151 in *Np*Xyn11A (Ile132 in *Ev*Xyn11^TS^) at −3 subsite; Tyr98 (Tyr80 *Ev*Xyn11^TS^) and Trp100 (Trp82 *Ev*Xyn11^TS^) at −2 subsite; Pro149 (Pro130 *Ev*Xyn11^TS^) and Phe158 (Phe138 *Ev*Xyn11^TS^) at −1 subsite; Tyr115 (Tyr91 *Ev*Xyn11^TS^) and Gln160 (Gln140 *Ev*Xyn11^TS^) at +1/−1 subsites; and Pro125 (Pro101 *Ev*Xyn11^TS^) at +1 subsite. Two additional highly conserved amino acid residues are found in the binding site of *Ev*Xyn11^TS^: Ser131 at the −3 subsite and Arg126 at the −1 subsite. In *Np*Xyn11A, the corresponding positions are occupied by Thr150 and His146.

The active site cleft (AS) is almost six times larger in *Np*Xyn11A than in *Ev*Xyn11^TS^. It encompasses 41 residues with a volume of 461.24 Å^3^ and an area of 521.62 Å^2^, while the active site cleft of *Ev*Xyn11^TS^ possesses only 23 residues, a volume of 77.12 Å^3^, and an area of 173.27 Å^2^. The larger and extended binding cleft of *Np*Xyn11A was shown to play an important role in the unusually high activity displayed by this enzyme [17]. Indeed, these active site features may facilitate the access or release of the substrate or product while better accommodating xylose units in each of its subsites. Furthermore, the wider cleft of *Np*Xyn11A might also facilitate the recognition and interaction with decorated xylans and, consequently, their hydrolysis.

**Figure 12:**
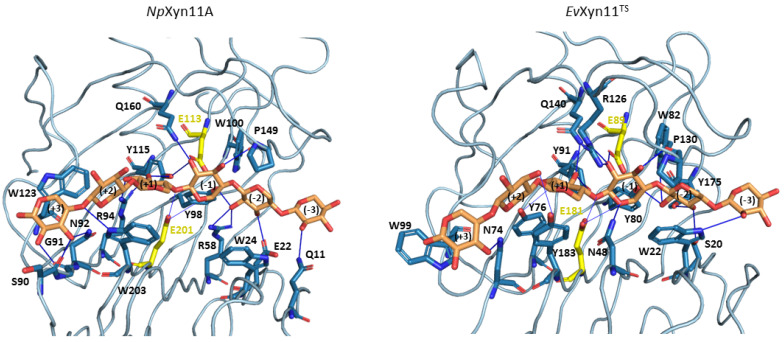
Noncovalent enzyme/xylohexaose contacts found with the Plip web-server on the equilibrated structure structures of *Np*Xyn11A and *Ev*Xyn11^TS^. Amino acid residues (colored in blue) in interaction with the xylohexaose (colored in orange) are shown in sticks. Catalytic amino acid residues are colored in yellow. Hydrogen bonds are shown with blue lines.

The xylohexaose (X6) adopts a similar conformation in the active site of both enzymes and is maintained in the binding cleft all along the MD simulations. As hydrogen bonds and stacking interactions provide the main ligand binding strengths, these noncovalent interactions have been evaluated using the PLIP web-server. To consider the most catalytically favorable conformation of xylohexaose in the binding site, we firstly chose to analyze the interactions in the 3D structure of *Np*Xyn11A/X6 and *Ev*Xyn11^TS^/X6 generated after the equilibration phase. Different interactions are presented in Figure 12, where we display the initial equilibrated configuration of the respective enzymes in complex with xylohexaose. There is a total of 20 different hydrogen bonding interactions with xylohexaose in *Np*Xyn11A, versus 16 in *Ev*Xyn11^TS^. The list of residues involved in hydrogen-bonding interactions with X6 in each enzyme is given in Table 6. These residues, distributed from −3 to +3 subsites of the binding cleft, include several highly conserved amino acid residues, previously mentioned (Tyr88, Trp100, Pro149, and Tyr115 in *Np*Xyn11A; Tyr80, Trp82, Pro130, and Tyr91 in *Ev*Xyn11^TS^). In addition to hydrogen bonds, the xylohexaose is also stabilized in the binding site by stacking interactions involving the aromatic amino acid residues: Trp24, Trp203, and Trp123 in *Np*Xyn11A; Trp22, Tyr183, and Trp99 in *Ev*Xyn11^TS^ at the subsites −2, +2, and +3, respectively.

The density of the enzyme–xylohexaose hydrogen bond network is relatively similar in the central subsites (−2 to +2) of both enzymes. However, subsite +3 shows a sparser network in *Ev*Xyn11^TS^ than in *Np*Xyn11A. In *Np*Xyn11A, the loop connecting the β-strands at subsite +3 is 4 residues longer than in *Ev*Xyn11^TS^ and allows more interactions with the xylose unit. The backbone carbonyl groups of Ser90 and Gly91 form hydrogen bonds with the hydroxyl groups of the xylose at subsite +3, in addition to the polar interaction involving the side chain of Asn92. In contrast, in *Ev*Xyn11^TS^, the loop is shorter, and only Asn74 makes a polar interaction with the xylose unit at subsite +3. While the side-chain of Gln10 (the first residue of a β-strand in the *N*-terminal domain of *Ev*Xyn11^TS^) points away from the xylohexaose binding site, the side-chain of the corresponding Gln11 in *Np*Xyn11A points towards the binding site and is perfectly oriented to make a polar contact with the xylose unit at subsite −3. The difference in the distal glycone (−3) and aglycone (+3) subsites of the extended cleft of *Np*Xyn11A might explain the high catalytic activity displayed by this enzyme compared to *Ev*Xyn11^TS^.

To go further in the investigation of enzyme–substrate interactions, the intermolecular hydrogen bonds formed between the enzymes and the xylohexaose substrate were also monitored during the first 100 ns of the respective MD trajectories at 310 K. Table 7 shows the frequency of occurrence of the intermolecular hydrogen bonds established between *Np*Xyn11A and xylohexaose, and between *Ev*Xyn11^TS^ and xylohexaose, respectively, when this frequency exceeds 10%.

Over the course of the MD trajectories, we observe the formation of only one additional type of intermolecular hydrogen bond in each complex compared to the ones initially observed in the equilibrated conformations. Indeed, a hydrogen bond is formed between the Asn54 and the xylose unit at subsite +1 of the *Np*Xyn11A cleft (with an occurrence frequency of 65%) and between the Asp105 located in the cord region and the xylose subunit in the subsite +2 of the *Ev*Xyn11^TS^ cleft (with a frequency of 28%) (Table 7). In the initial *Ev*Xyn11^TS^ structure, the Asp105 is located at a distance of 7 Å from the ligand. This observation confirms that a conformational rearrangement of this flexible region is required to allow this amino acid to interact with the substrate in the *Ev*Xyn11^TS^/X6 complex. It is probable that the binding of the ligand triggers this conformational change.

Some interactions, observed in the initial configurations, are present in less than 10% of the 100 ns MD of both *Np*Xyn11A and *Ev*Xyn11^TS^. In *Np*Xyn11A, this is especially the case for the side-chains of Arg58 and Trp100, which initially interact with the xylose unit at subsite −2; Gln160, which initially interacts with the xylose at subsite −1; Tyr115, which initially interacts with the xylose at subsites −1 and +1; and Arg94, which initially interacts with the xylose at subsite +1. In *Ev*Xyn11^TS^, this is the case for the side-chain of Trp22, which initially interacts with the xylose at subsite −3; Ser20 and Trp82, which initially interact with the xylose at subsite −2; Tyr91, which initially interacts with the xylose at subsite +1; and finally, Tyr183, which initially interacts with the xylose at subsite +2.

Compared with *Np*Xyn11A, the interaction of the substrate in the −3 subsite (glycone) is less stable in *Ev*Xyn11^TS^. As hydrogen interactions involving the residues of the distal glycone subsite (−3) are crucial for the binding of X6 substrate, the loss of interaction identified in *Ev*Xyn11^TS^ might contribute to its lower catalytic activity. At subsite −2, the conserved tyrosine in GH-11 xylanases; Tyr98 and Tyr80 in *Np*Xyn11A and *Ev*Xyn11^TS^, respectively; in addition to Glu22 in *Np*Xyn11A and Tyr175 in *Ev*Xyn11^TS^, make polar interactions with the substrate along MD simulations with an occurrence frequency of 21%, 67%, 43% and 80% respectively. The lower occurrence frequency of these interactions in *Np*Xyn11A may be explained by the bigger *Np*Xyn11A active site cleft, especially in the glycone region, thus enabling the substrate to interact with more residues over the course of the simulation. Furthermore, the presence of a tryptophan residue at position 24 in *Np*Xyn11A and at position 22 in *Ev*Xyn11^TS^ likely promotes a stacking interaction of the indole ring with the xylose moiety at subsite −2. At subsite −1, the highly conserved proline in GH-11 xylanases, found at position 149 in *Np*Xyn11A 130 in *Ev*Xyn11^TS^, and located in the thumb loop, is involved in forming a conserved pattern of HB interactions in more than 76% of both enzymes MD trajectories (Table 7). Arg126 in *Ev*Xyn11^TS^, which is also located in the thumb region close to this proline in *Ev*Xyn11^TS^, makes a polar contact with the xylose unit at subsite −1 (Figure 12). Histidine (His146) residue is found at this position in *Np*Xyn11A. It has been suggested that this residue could form polar contacts with surrounding residues [17]. As observed in the equilibrated conformations, the substrate is better stabilized with more polar contacts at subsite +3 of *Np*Xyn11A than in *Ev*Xyn11^TS^. The substrate binding to the distal glycone and aglycone subsites is thus weaker in *Ev*Xyn11^TS^. By contrast, the topology of the *Np*Xyn11A cleft allows it to better accommodate and make more polar interactions with the xylose units at the −3 and +3 subsites, which may contribute to the highly enhanced activity of *Np*Xyn11A in comparison to *Ev*Xyn11^TS^.

The analyses of the unusually active *Np*Xyn11A and hyper-thermostable *Ev*Xyn11^TS^ in complex with xylohexaose revealed details on the molecular determinants playing a key role on xylohexaose binding interactions, which could be transposed to other xylanases in the GH-11 family.

## 4. Conclusions

In this study, we investigated the dynamic properties of two different xylanases from the GH-11 family: the particularly active GH-11 xylanase from *Neocallimastix patriciarum*; *Np*Xyn11A [17]; and the thermostable mutant of environmentally isolated GH-11 xylanase, *Ev*Xyn11^TS^ [18].

The analysis of molecular flexibility combined with the monitoring of specific structural and geometrical properties revealed that *Ev*Xyn11^TS^ is more tightly packed and shows a higher number of stable intramolecular interactions. Its shorter loops, as well as the presence of a disulfide bridge at the *N*-ter region and stable salt bridges, may also explain its excellent stability. However, this overall rigidity contrasts with the greater conformational variability of the thumb region. As previously analyzed on related thermostable GH-11 xylanases, this highly mobile thumb may facilitate the access and release of the ligand into the otherwise relatively narrow and short catalytic cleft of *Ev*Xyn11^TS^ [48]. The thumb is then stabilized in a catalytic-competent conformation in the presence of the ligand.

Instead, *Np*Xyn11A has more flexible regions. At a high temperature (500 K), MD simulations show that *Np*Xyn11A tends to unfold after a few ns while *Ev*Xyn11^TS^ withstands this high temperature for 100 ns at least. The *N*-terminal region of *Np*Xyn11A lacks *Ev*Xyn11^TS^’s disulfide bridge, and its loose hydrogen network also leads to unreliable salt bridges and higher flexibility in simulations. We observed that the cord region presents very high flexibility in the free-enzyme form but seems to be stabilized when in complex with xylohexaose. A relatively high flexibility of the palm loop and the helix loop is also observed in *Np*Xyn11A, even in its complex form. The cross-correlation analysis confirmed the flexibility of these regions and showed that they are correlated in *Np*Xyn11A, especially in the palm loop or the B3-A5 loop region. These correlations are absent in *Ev*Xyn11^TS^. These regions represent clear potential stabilization hotspots.

As a surprise, compared to *Ev*Xyn11^TS^, the thumb region of *Np*Xyn11A seems moderately flexible in both its free-enzyme and complex forms, probably because of a strong salt bridge. This suggests that *Np*Xyn11A possesses a thumb conformation that is already competent for catalysis in its unbound form. We hypothesize that the large catalytic cleft of *Np*Xyn11A, with its better organized subsites at the extremities of the cleft, may facilitate the access, binding, and release of the ligand. These steps may be limiting in *Ev*Xyn11^TS^ because of its narrower and shorter cleft, and this may explain the need for a highly mobile thumb [48]. *Np*Xyn11A can instead benefit from what looks like a preconfigured thumb conformation. Eventually, the relatively rigid thumb region and the larger catalytic site cleft of *Np*Xyn11A seem to play a major role on the activity of this enzyme. These MD-simulation-based findings are also consistent with the very recent experiments on a bioengineered *Bacillus circulans* GH-11 family xylanase with increased thumb flexibility, which led to reduced hydrolysis activity [49].

These analyses bring to light an attractive redesign strategy for improving xylanases for applications that require high catalytic activity combined with excellent thermostability. A feasible target would be to convey several of the identified thermostabilizing traits of *Ev*Xyn11^TS^ to *Np*Xyn11A, by acting on regions located further from the active site such as the *N*-ter region, the loops connecting the fingers, the palm loop, as well as the helix and the B3-A5 loop regions. These regions seem to be less stable than in the hyper-thermostable *Ev*Xyn11^TS^ and offer potential stabilization hotspots. The converse strategy that would try to improve *Ev*Xyn11^TS^ activity would instead require extensive remodeling of the catalytic cleft and of the thumb mobility, which seems far more challenging.

## Figures and Tables

**Table 1:** Definition of structural regions of the *Np*Xyn11A and *Ev*Xyn11^TS^ enzymes by their respective residue number.

Regions	*Np*Xyn11A	*Ev*Xyn11^TS^
Fingers	4–62,76–95,199–216	4–53,63–78,179–191
Thumb; Thumb loop	137–167; 149–155	117–147; 130–135
Palm; Palm loop	96–100,113–121,195–199; 102–113	79–83,89–95,172–178; 84–88
Helix; Helix loop	176–186; 187–194	155–166; 167–171
Cord	122–129	96–106
Loop B3-A5	63–75	54–62

**Table 2:** Number of hydrogen-bonding interactions in *Np*Xyn11A and *Ev*Xyn11^TS^. Intramolecular static, dynamic, and the number of enzyme–solvent hydrogen bonds are given (standard errors are in parentheses and not given for dynamic HB and HBs with solvent as these measures depend on the simulation length).

System	T	Static HB	Dynamic HB	HBs with Solvent
*Np*Xyn11A	310 K	0.57 (0.002)	17.01	1473.03
	340 K	0.56 (0.003)	20.87	1356.64
*Ev*Xyn11^TS^	310 K	0.62 (0.002)	16.53	1228.94
	340 K	0.60 (0.002)	18.17	1125.52
*Np*Xyn11A/X6	310 K	0.56 (0.002)	16.54	1427.75
	340 K	0.56 (0.002)	19.75	1327.13
*Ev*Xyn11^TS^/X6	310 K	0.62 (0.003)	14.62	1115.26
	340 K	0.61 (0.003)	17.37	1068.98

**Table 3:** Occurrence fraction in percentage of salt bridges identified in MD simulations (standard errors are in parentheses).

Enzyme	Salt Bridge	Free		Complex/X6	
		310 K	340 K	310 K	340 K
*Np*Xyn11A	Asp119-Lys168	38.9 (3.7)	-	33.8 (5.4)	35.8 (1.3)
	Asp126-Lys140	92.3 (1.9)	68.5 (7.7)	90.2 (3.4)	91.9 (1.0)
	Asp145-Lys159	82.6 (1.2)	81.8 (0.7)	78.5 (2.9)	77.4 (2.0)
	Asp210-Lys43	54.6 (0.7)	54.71 (2.1)	55.3 (1.1)	60.4 (0.6)
	Glu118-Arg169	98.7 (0.3)	96.9 (0.3)	98.8 (0.4)	96.9 (0.8)
	Glu182-Lys185	68.9 (4.9)	72.0 (2.9))	78.2 (2.2)	72.8 (1.7)
	Glu22-Lys13	54.9 (1.3)	45.6 (0.7)	48.4 (1.9)	33.1 (2.1)
	Glu201-Arg94	38.1 (10.8)	46.50 (11.0)	-	-
*Ev*Xyn11^TS^	Asp161-Arg60	95.7 (2.5)	97.7 (0.4)	99.6 (0.01)	97.7(1.4)
	Asp190-Arg38	99.7 (0.1)	99.20 (0.4)	99.8 (0.1)	99.6 (0.1)
	Asp94-Arg149	99.9 (0.1)	98.9 (0.2)	99.9 (0.02)	99.5 (0.2)
	Glu89-Arg126	94.6 (4.9)	96.1 (3.9)	-	-

**Table 4:** Values of SASA of each system in their free-enzyme and enzyme–substrate complex forms at 310 K and 340 K (standard errors are in parentheses).

System	T	SASA (Å^2^)
*Np*Xyn11A	310 K	8714.9 (10.8)
	340 K	8840.3 (33.8)
*Ev*Xyn11^TS^	310 K	7224.6 (46.6)
	340 K	7368.1 (14.5)
*Np*Xyn11A/X6	310 K	8549.5 (47.1)
	340 K	8430.2 (34.5)
*Ev*Xyn11^TS^/X6	310 K	6664.35 (14.9)
	340 K	6900.1 (44.5)

**Table 5:** Geometrical and topological properties of each enzyme’s active site.

Enzyme	Nb Residues	Volume (AS, Å^3^)	Area (AS, Å^2^)
*Np*Xyn11A	41	461.24	521.68
*Ev*Xyn11^TS^	23	77.12	173.27

**Table 6:** List of residues involved in hydrogen bonding with X6 in *Np*Xyn11A and *Ev*Xyn11^TS^ equilibrated structures. The nature of the hydrogen bond partner, a side-chain (SC), or Backbone (BB) atom is indicated as well as the subsite of the interaction in the cleft.

*Np*Xyn11A/X6			*Ev*Xyn11^TS^/X6		
Gln11	SC	−3	Trp22	SC	−3
Glu22	SC	−2	Ser20	SC	−2
Arg58	SC	−2	Tyr80	SC	−2
Tyr98	SC	−2	Trp82	SC	−2
Trp100	SC	−2	Tyr175	SC	−2
Glu113	SC	−1	Asn48	SC	−1
Pro149	BB	−1	Glu89	SC	−1
Gln160	SC	−1	Arg126	SC	−1
Glu201	SC	−1/+1	Pro130	BB	−1
Tyr115	SC	−1/+1	Glu181	SC	−1/+1
Arg94	SC	+1	Tyr76	SC	+1
Trp203	SC	+2/+3	Gln140	SC	+1
Ser90	BB	+3	Tyr91	SC	+1
Gly91	BB	+3	Tyr183	SC	+2
Asn92	SC	+3	Asn74	SC	+3

**Table 7:** Percentage of occurrence of the intermolecular hydrogen bonds between *Np*Xyn11A (resp. *Ev*Xyn11^TS^) amino acid residues and the xylohexaose calculated from MD simulations at 310 K. The localization of the amino acid residues in the subsites of the cleft is indicated.

*Np*Xyn11A/X6 310 K			*Ev*Xyn11^TS^/X6 310 K		
**HB_inter_**	**Percentage**	**Subsite**	**HB_inter_**	**Percentage**	**Subsite**
Gln11−X6	22%	−3	Tyr80−X6	67%	−2
Glu22−X6	43%	−2	Tyr175−X6	80%	−2
Tyr98−X6	21%	−2	Asn48−X6	51%	−1
Glu113−X6	77%	−1	Glu89−X6	95%	−1
Pro149−X6	79%	−1	Arg126−X6	29%	−1
Asn54−X6	65%	+1	Pro130−X6	77%	−1
Trp203−X6	10%	+2/+3	Tyr76−X6	24%	+1
Ser90−X6	58%	+3	Gln140−X6	30%	+1
Asn92−X6	29%	+3	Asp105−X6	28%	+2
			Asn74−X6	38%	+3

## Data Availability

Data is contained within the article.
